# Augmented stabilization of a rare geriatric “open book”-injury, using a cement augmented internal fixator – a case report

**DOI:** 10.1016/j.tcr.2026.101327

**Published:** 2026-04-22

**Authors:** Alexa Stansell, Antonius Pizanis, David Osche, Marcel Orth, Emmanouil Liodakis, Tim Pohlemann, Tobias Fritz

**Affiliations:** Saarland University Medical Center, Department for Trauma, Hand and Reconstructive Surgery, Kirrbergerstr. 100, 66421, Homburg, Germany

**Keywords:** Open book injury, Pelvic ring fracture, Osteoporotic bone, Internal fixator, Minimally invasive stabilization pelvis

## Abstract

**Background:**

Pelvic ring injuries in elderly patients, particularly in the context of low-energy trauma, typically involve posterior ring structures due to underlying osteopenia or osteoporosis. However, symphyseal disruptions are extremely rare in this population. Pelvic ring injuries in elderly patients are increasingly common due to demographic changes, often resulting from low-energy falls. We present a unique case of a 77-year-old morbidly obese female with a Type B1.1 pelvic ring injury (APC-2, according to Young & Burgess classification), characterized by an open book lesion with no posterior fracture. Given the patient's body habitus and reduced bone quality, a minimally invasive internal fixator spine system was implanted and augmented with polymethylmethacrylate (PMMA) cement to enhance fixation strength and allow early mobilization.

**Case presentation:**

A 77-year-old female (BMI: 36 kg/m^2^) presented with a rare isolated Type B1.1 pelvic ring injury (Fig. 1A) following a domestic fall. After emergency stabilization using an external fixator, in a secondary definitive surgery a subcutaneous internal fixator was implanted bilaterally in a minimally invasive fashion. To improve fixation in the osteoporotic supra-acetabular bone, the screws were cement-augmented using PMMA. The patient showed good postoperative recovery, early mobilization, and stable radiographic outcomes at 6 weeks follow-up.

**Conclusion:**

This case illustrates the feasibility and benefit of cement-augmented internal fixator of the pubic symphysis with a rare case of an “open-book” injury in elderly, obese female patient. Cement augmentation may offer additional pull-out strength in patients with reduced bone quality, facilitating early mobilization and avoiding complications related to implant loosening.

## Introduction

A 77-year-old female patient with a BMI of 36 kg/m^2^ presented to our institution after sustaining a splits-like fall on a slippery floor when she was cleaning her house. She reported severe anterior pelvic pain and was unable to bear weight on her lower limbs. Clinical examination revealed tenderness over the pubic symphysis without signs of neurological deficit.

**Fig. 1:**
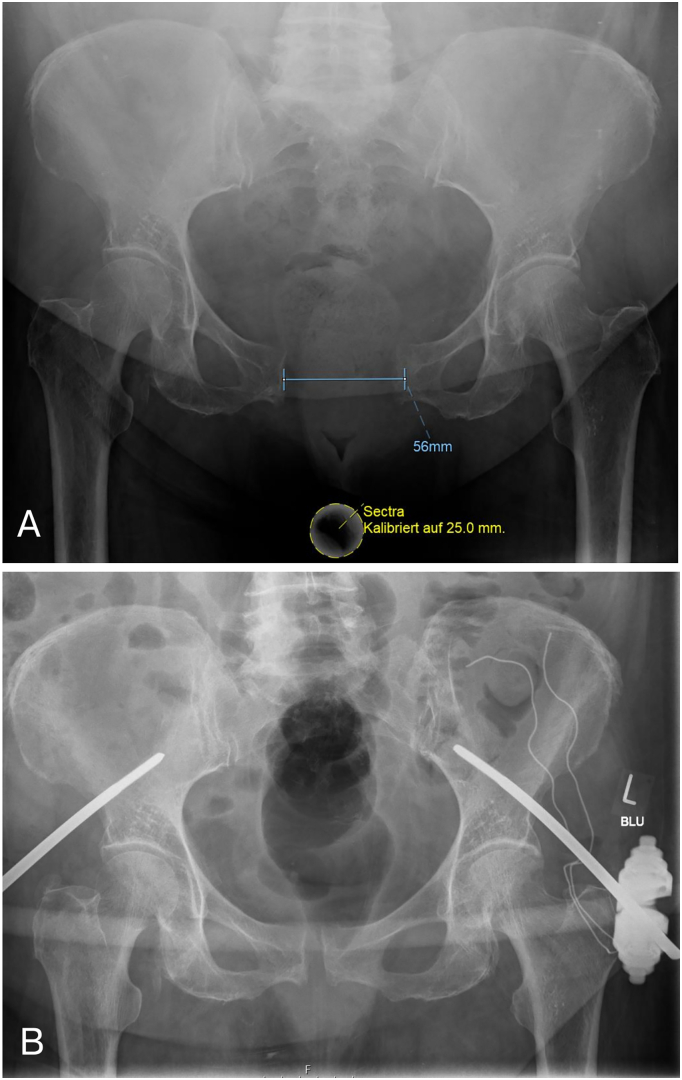
A: Shows the posttraumatic X-ray, a.p. of the pelvis with a diastasis of 56 mm. B: Shows the postoperative X-ray, a.p. after emergency stabilization using an external fixator of the pelvis.

Radiographs and computed tomography (CT) scans demonstrated a Type B1.1 pelvic ring injury characterized by a pubic symphysis diastasis of 56 mm with intact posterior pelvic ring structures (“open-book”-injury) (Fig. 1A). Bone quality was assessed as osteoporotic. Initially an emergency treatment using an external fixator of the pelvis was conducted (Fig. 1B).

**Fig. 2:**
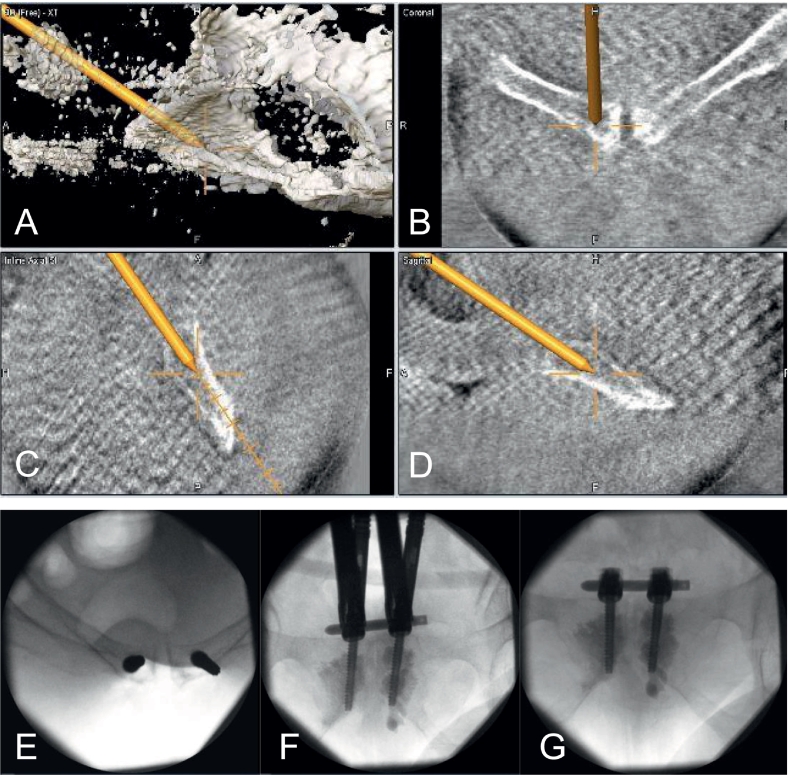
A-D Shows the intraoperative used 3D-navigation to optimize the Schanz screw placement. E: Shows the placed Schanz-screws in an inlet view before cement augmentation F: Shows the reduction maneuver for alignment of the vertical and horizontal dislocation of the pubic symphysis. G: Shows the final internal fixator construct.

Due to the patient's morbid obesity and poor bone stock, a minimally invasive stabilization technique was chosen for definitive stabilization of anterior pelvic ring. Using an optical navigation system (Brainlab, Munich, Germany) (Fig. 2A-D) and fluoroscopic guidance (Fig. 2E-G), a bilateral subcutaneous internal fixator was implanted. To enhance screw purchase in the osteoporotic supra-acetabular bone, polymethylmethacrylate (PMMA) cement augmentation was performed at the screw sites.

**Fig. 3:**
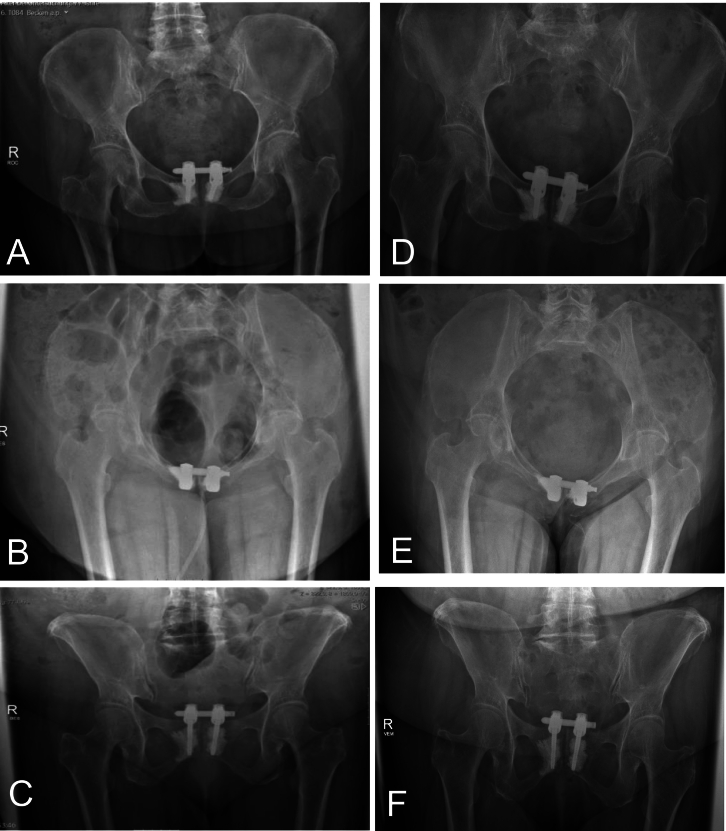
A-C Shows the postoperative X-ray, a.-p. (A), inlet (B) and outlet (C) view. D-F Shows the follow-up X-rays 7 years postoperative, a.-p. (D), inlet (E) and outlet (F) view.

The surgery was completed without complications. Postoperative imaging confirmed adequate reduction of the anterior pelvic ring and correct implant positioning (Fig. 3). The patient was mobilized with full weight bearing starting on the second postoperative day. At six weeks follow-up, the patient demonstrated improved pain control and stable radiographic findings (Fig. 3D-F). At three months, full weight bearing without assistance was possible, and no implant-related complications or neurological symptoms were noted.

## Surgical technique

To stabilize the pubic symphysis, the USS MIS system was applied (J&J MedTech, Umkirch, Germany). The patient was positioned in supine position and a DAB was placed on the left iliac wing for intraoperative 3D-navigation (Brainlab, Munich, Germany) (Fig. 2A-D). After the acquisition of an intraoperative 3D-scan, a 3 cm supra symphyseal incision was performed. Using the navigation system two guide-wires were placed parallel to the pubic symphysis from cranial to caudal. Then two cannulated 5 mm Schanz-screws (J&J MedTech, Umkirch, Germany) were placed over the guide-wires and augmented with PMMA (Kyphon, Medtronic) (Fig. 2E). After polymerization the fracture clamps and linked by a 6 mm titanium rod (J&J MedTech, Umkirch, Germany) were placed on the Schanz-screws as utilized for spine surgery. Both Schanz-screws were pulled laterally and aligned vertically to allow a caudal compression of the symphysis, and the bolts were tightened. A second reduction maneuver was performed, after loosening the adjunct bolts for lateral compression, to also achieve cranial compression of the symphysis (Fig. 2F). Thereafter, the Schanz-screws were cut and the soft tissue closed.

The final fluoroscopic images (Fig. 2G), additional postoperative radiographs (Fig. 3A-C) and a CT-scan demonstrated an anatomical reduction and correct implant position.

In the clinical follow up 6 weeks postoperative, no secondary implant loosening or secondary dislocation were detected. The patient presented again for follow up 7 years postoperative in our department. The patient reported no pain and no symptoms of soft tissue irritation, even over this long time period. Furthermore, the X-rays showed no signs of loosening of implant breakage (Fig. 3D-F). She reported no loss of mobility compared to the preinjury level at this time point.

## Discussion

The treatment of pelvic ring injuries in morbidly obese and elderly patients poses unique challenges due to a combination of biomechanical loading, poor bone quality, and increased risk of soft tissue complications [15], [16], [17], [18], [19]. Minimally invasive and percutaneous surgical techniques have are in focus of current research as a soft tissue–sparing alternative to the current gold standard of conventional open reduction and plating techniques [1], [2], [3]. However, none of the novel described alternative minimally invasive stabilization techniques (e.g., Symfix, internal fixator, INFIX, Pelvic Bridge, Cannulated Screws) have replaced the current plate osteosynthesis so far [4], [5], [6]. Whilst the number of fragility fractures of the pelvis has risen in the last years [15], [16], [17], [18], [19], only few cases of “open-book”-injuries in the elderly has been described [7], [15], [16], [17], [18], [19]. This injury pattern is typically seen in younger patients or in the context of high-energy trauma [17]. In this case, the unusual combination of advanced age, obesity, and anterior-only instability required a fixation technique that balanced biomechanical strength with minimal invasiveness. Recent reports showed that a laparoscopic approach for symphyseal plating is feasible [8], however, most orthopaedic surgeons are more familiar using minimally invasive spine systems compared to laparoscopic techniques. Subcutaneous internal fixator techniques have been used with reduced soft tissue damage due to dissection, thereby minimizing the risk of wound healing complications and infection [7], [9] — particularly relevant in obese individuals with large skin folds and reduced perfusion [9], [10]. Additionally, the biomechanical properties of the internal fixator system have shown to provide sufficient stabilization for anterior pelvic ring disruptions, allowing for early mobilization [9], [11], [12].

However, the effectiveness of screw anchorage in osteoporotic bone remains a critical concern, particularly in elderly patients. To address this, we employed PMMA cement augmentation at the parasymphyseal screw sites, a technique well established in spinal and periacetabular fixation for enhancing screw purchase [13]. Cement augmentation in this context not only increased primary stability but also reduced the risk of screw loosening under high mechanical stress [14]. This enabled early postoperative mobilization and likely contributed to the uneventful healing course observed in our patient [14]. No neurological deficits or implant-related complaints were noted during follow-up.

Our case further supports the expanding role of minimally invasive, subcutaneous fixation methods in complex patient populations. The successful use of a cement-augmented internal symphysis fixator in an elderly, obese patient with osteoporotic bone and a rare anterior-only pelvic injury suggests that this technique can be considered even in atypical fracture patterns, provided that the posterior ring remains intact and technical precautions are observed.

## Conclusion of discussion

This case illustrates that a cement-augmented internal fixator of the pubic symphysis can provide sufficient stabilization for isolated anterior pelvic ring injuries in elderly, morbidly obese patients. The technique allowed for early mobilization, minimized soft tissue damage, and addressed challenges related to osteoporotic bone. Cement augmentation may represent a valuable adjunct in selected patients where screw stability is of concern.

## CRediT authorship contribution statement

**Alexa Stansell:** Writing – original draft. **Antonius Pizanis:** Visualization, Supervision, Investigation, Conceptualization. **David Osche:** Writing – review & editing. **Marcel Orth:** Writing – review & editing, Validation. **Emmanouil Liodakis:** Writing – review & editing. **Tim Pohlemann:** Writing – review & editing. **Tobias Fritz:** Writing – review & editing, Writing – original draft, Visualization, Validation, Supervision, Methodology, Investigation, Data curation, Conceptualization.

## Disclaimer

The patient consented in use of the presented data for scientific publication.

## Declaration of competing interest

The authors declare that they have no known competing financial interests or personal relationships that could have appeared to influence the work reported in this paper.
